# Comparative effectiveness of enoxaparin versus unfractionated heparin for 30- and 90-day mortality in sepsis patients without severe renal dysfunction: a retrospective cohort study

**DOI:** 10.3389/fmed.2026.1886512

**Published:** 2026-10-26

**Authors:** Zhe Xue, Rubing Guo, Xiaolin Lou, Yuanshu Tian, Jingjing Tong, Wei Zhao

**Affiliations:** 1 School of Public Health, Gansu University of Traditional Chinese Medicine, Lanzhou, China; 2 Department of Clinical Laboratory, China-Japan Friendship Hospital, Beijing, China; 3 Department of Infectious Diseases, China-Japan Friendship Hospital, Beijing, China

**Keywords:** enoxaparin, mortality, retrospective cohort study, sepsis, survival analysis, unfractionated heparin

## Abstract

**Background:**

Although enoxaparin is known to be superior to UFH for thromboprophylaxis, the comparative effect of enoxaparin versus UFH on mortality in sepsis remains unknown. To address this gap, we compared the two agents in sepsis patients without severe renal impairment, thereby minimizing confounding due to differential drug clearance.

**Methods:**

In this retrospective cohort study, we analyzed data from the eICU Collaborative Research Database. We included adult patients with sepsis and no severe renal impairment (eGFR ≥ 30 mL/min/1.73 m^2^) who received prophylactic anticoagulation with either enoxaparin or UFH. The primary outcomes were 30-day ICU mortality and 90-day in-hospital mortality. Survival analyses were performed using Kaplan–Meier estimates and Cox proportional hazards models. Sensitivity analyses were conducted using inverse probability of treatment weighting.

**Results:**

A total of 2,341 patients were enrolled (1,260 in the enoxaparin group and 1,081 in the UFH group). After adjusting for confounders, multivariate Cox regression showed that the hazard ratio (HR) for enoxaparin versus UFH was 0.94 (95% CI: 0.67–1.32, *P* = 0.726) for 30-day ICU mortality and 0.86 (95% CI: 0.66–1.12, *P* = 0.271) for 90-day in-hospital mortality. Subgroup analyses showed no significant heterogeneity across predefined strata of age, sex, or BMI (all P for interaction >0.05). These findings were supported by inverse probability of treatment weighting analysis.

**Conclusions:**

In this cohort of sepsis patients without severe renal impairment, enoxaparin was associated with numerically lower but not statistically significant hazard ratios for both 30-day ICU and 90-day in-hospital mortality compared with UFH. These findings do not establish the superiority of either agent. Larger, prospective studies are warranted to confirm the observed associations.

## Introduction

Sepsis is a systemic inflammatory response syndrome triggered by infection and a primary cause of multiple organ dysfunction syndrome and septic shock. In intensive care units (ICUs), it is associated with particularly high incidence and mortality rates (1). Globally, sepsis affects millions of patients annually, with mortality rates exceeding 25%. Furthermore, its incidence continues to rise, making it a persistent and serious global health challenge (2).

Critically ill patients with sepsis are at high risk for deep vein thrombosis (DVT) and pulmonary embolism (PE). The incidence of ICU-acquired DVT can reach 10%, while that of PE is approximately 2%–4% (3, 4). Heparin-based thromboprophylaxis reduces the incidence of both DVT (RR 0.51 [95% CI, 0.41–0.63]; *p* < 0.0001) and PE (RR 0.52 [95% CI 0.28–0.97]; *p* = 0.04) (5). Low-molecular-weight heparin (LMWH), such as enoxaparin, has been clinically proven to reduce venous thromboembolism (VTE). Compared with UFH, enoxaparin significantly lowers the incidence of VTE in hospitalized medical patients (RR 0.37 [97.6% CI, 0.22–0.63]; *p* < 0.001) (6). Consequently, the 2021 sepsis treatment guidelines recommend pharmacologic VTE prophylaxis, specifying LMWH over UFH when no contraindications exist (7). However, this is a conditional recommendation based on low-certainty evidence. Notably, the guideline panel's literature review found no randomized controlled trial evidence directly comparing LMWH with UFH in septic patients.

Evidence suggests that both UFH and LMWH may reduce mortality in sepsis. UFH has been associated with a lower 28-day mortality risk (HR 0.70, 95% CI 0.56–0.87) (8–10), and LMWH has shown a similar benefit (*p* = 0.026) (11). However, these findings are derived from separate comparisons against placebo or no heparin, rather than from direct head-to-head comparisons between the two agents. Consequently, whether enoxaparin confers a mortality advantage over UFH in sepsis remains unknown—a question that the current guideline recommendation, focused primarily on VTE prevention, does not address.

A major barrier to obtaining such direct comparative evidence is the confounding effect of acute kidney injury (AKI), a common complication in sepsis (12, 13). Because enoxaparin is cleared renally, while UFH is cleared via both liver and reticuloendothelial pathways, AKI differentially prolongs the half-life of enoxaparin to a much greater extent than that of UFH (14). This disparity can lead to supratherapeutic anti-Xa levels and an increased bleeding risk with standard enoxaparin doses in patients with renal impairment (15). More importantly, it also means that in an unselected septic population, any observed mortality difference between the two drugs could be driven not by their intrinsic pharmacologic effects, but by the differential impact of renal dysfunction on drug accumulation and safety.

To minimize this confounding and isolate the comparative effect of the two agents on mortality, we restricted our analysis to septic patients without severe renal impairment (eGFR ≥ 30 mL/min/1.73 m^2^). By doing so, we aimed to obtain a comparison of enoxaparin versus UFH with reduced confounding from renal function.

## Methods

### Data source

This retrospective cohort study used data from the eICU Collaborative Research Database (eICU-CRD), a publicly available, de-identified multicenter critical care database developed through a collaboration between Philips Healthcare and the MIT Laboratory for Computational Physiology. The eICU-CRD contains records of 200,859 ICU admissions from 208 U.S. hospitals that participated in the Philips eICU telemedicine program between 2014 and 2015. Available data include vital signs, laboratory results, medication use, APACHE IV scores, nursing plans, admission diagnoses, patient history, and treatment interventions. Because the database is de-identified, the identities of the participating hospitals are not publicly available. Access to the database is available via the official website (https://eicu-crd.mit.edu) after completion of required data use agreements. Further details are provided in the original publication (16).

### Study population

The initial cohort comprised 14,227 patients with a primary diagnosis of sepsis, identified by ICD-10 codes, at the time of ICU admission. After applying the inclusion criterion of an initial ICU stay of at least 24 h, 9,901 patients remained. Patients were excluded if they met any of the following criteria: (1) receipt of neither UFH nor enoxaparin; (2) concurrent use of UFH and enoxaparin; (3) administration of therapeutic-dose UFH or enoxaparin; (4) eGFR <30 mL/min/1.73 m^2^. Following these exclusions, 2,341 patients were included in the final analysis, of whom 1,081 received prophylactic-dose UFH alone and 1,260 received prophylactic-dose enoxaparin alone. The screening flowchart is presented in Figure 1.

**Figure 1:**
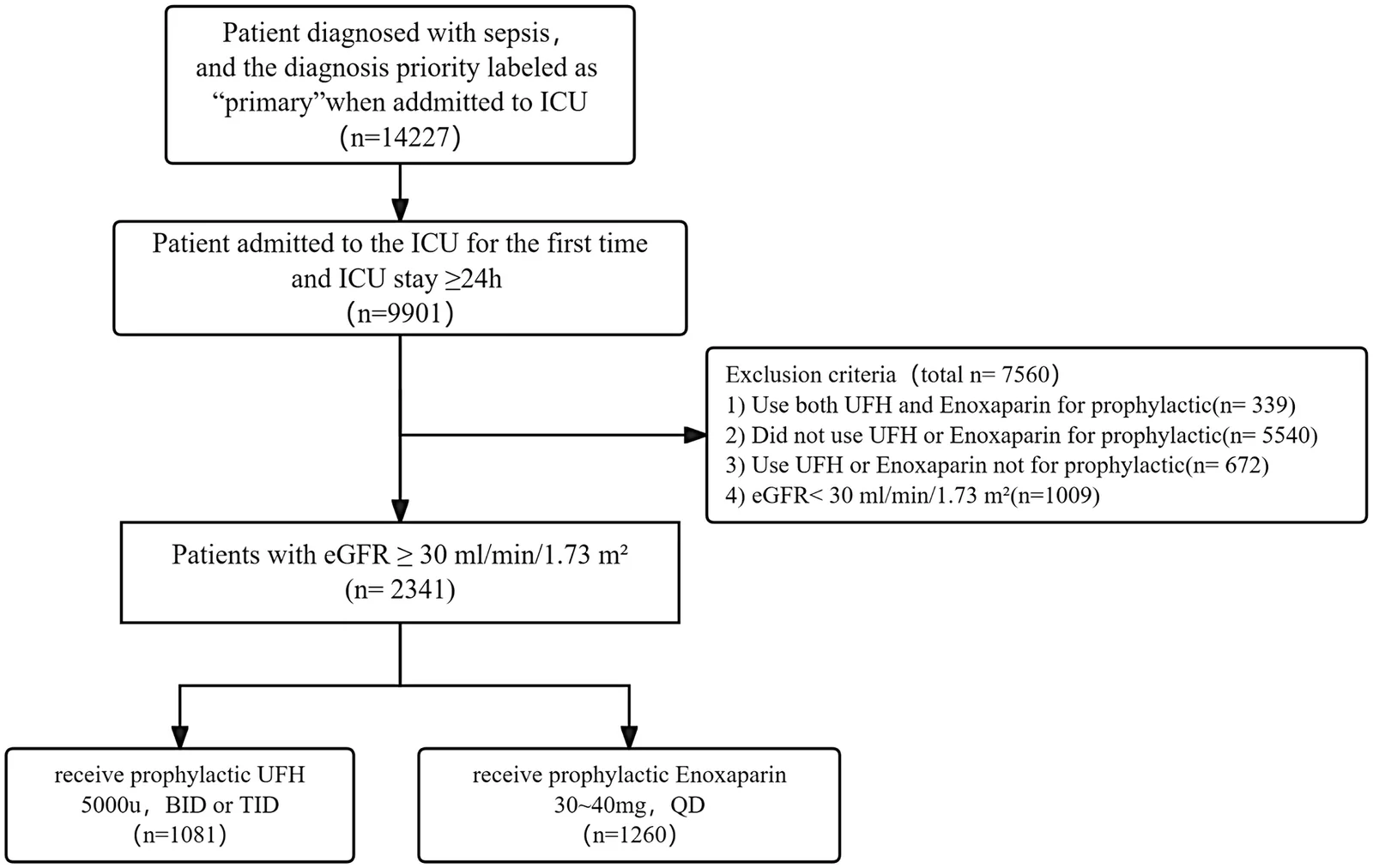
Flowchart of patient selection.

### Data extraction

Data extracted from the eICU-CRD included demographics, ICD-10-coded diagnoses, vital signs, and laboratory results. Baseline characteristics were defined as follows: height, weight, and APACHE IV score were taken from the first measurement after ICU admission; the lowest values of albumin, hemoglobin (Hb), and platelet count within the first 24 h were used; and mean values over the same 24-hour period were calculated for frequently monitored vital signs, including heart rate, respiratory rate, SpO₂, systolic/diastolic blood pressure (SBP/DBP), and body temperature.

### Exposure and outcomes

Patients were assigned to exposure groups based on prophylactic anticoagulation regimen: (1) the UFH group, receiving 5,000 units subcutaneously 2–3 times daily; and (2) the enoxaparin group, receiving 30–40 mg subcutaneously once daily. The primary outcome was all-cause mortality during follow-up, with in-hospital death as the endpoint. Mortality was assessed at 30 days after ICU admission and at 90 days after hospital admission.

### Statistical analysis

Normally distributed continuous variables are presented as mean ± standard deviation (SD) and compared using independent-samples t-test. Skewed continuous variables are presented as median (Q1, Q3) and compared using the Mann–Whitney U test. Categorical variables are expressed as frequency (percentage) and analyzed using the chi-square test. The primary outcome was assessed using Kaplan–Meier survival curves, and intergroup disparities were compared using the log-rank test. The association between anticoagulant type (enoxaparin vs. UFH) and mortality was evaluated using multivariate Cox proportional hazards regression with results presented as hazard ratios (HRs) with 95% confidence intervals (CIs). Three adjustment models were constructed: Model 1, unadjusted; Model 2, adjusted for sex, age, and body mass index (BMI); and Model 3, additionally adjusted for SpO₂, heart rate, APACHE IV score, albumin, bilirubin, BUN, creatinine, and lactate. Subgroup analyses were performed using Cox regression stratified by age, sex, and BMI, with the same covariate set as Model 3 (excluding the stratification variable). Interaction *P* values were calculated to assess effect modification across strata. To enhance robustness, we conducted an inverse probability of treatment weighting (IPTW) analysis. For the calculation of stabilized IPTW weights, propensity scores were first estimated using logistic regression with treatment assignment as the outcome and the Model 3 covariates as predictors. These scores were then used to derive the weights, which were incorporated into weighted Cox models. Covariate balance post-weighting was assessed using standardized mean differences (SMDs), with an SMD <0.1 considered adequate.

All tests were two-sided, with a *P* value < 0.05 considered statistically significant. All analyses were performed using R software (version 4.x).

## Results

### Baseline characteristics of the study population

Among the 2,341 sepsis patients included, 1,081 received prophylactic-dose UFH, and 1,260 received prophylactic-dose enoxaparin. Compared with the UFH group, patients in the enoxaparin group had significantly higher Hb, platelet, and temperature, but significantly lower mean age, APACHE IV score, BUN, creatinine, INR, and SpO₂ (all *P* < 0.05; Table 1).

**Table 1:** Baseline characteristics of patients.

Variable	Total (*n* = 2,341)		*P*-value
	UFH (*n* = 1,081)	Enoxaparin(*n* = 1,260)	
Age	67.00 (55.00, 79.00)	64.00 (52.00, 74.00)	<0.001
Sex, *n* (%)			0.465
Male	517 (47.83)	620 (49.21)	
Female	563 (52.08)	640 (50.79)	
BMI, kg/m^2^	26.41 (22.35, 31.77)	26.85 (22.37, 32.67)	0.294
APACHE IV	63.49 (49.55, 78.00)	61.00 (47.00, 75.66)	0.004
Albumin, g/dL	2.71 ± 0.66	2.68 ± 0.67	0.223
Bilirubin, mg/dL	0.73 (0.50, 1.12)	0.70 (0.50, 1.10)	0.113
Hb, g/dL	10.26 ± 2.10	10.62 ± 2.19	<0.001
Lactate, mmol/L	2.31 (1.60, 3.30)	2.21 (1.50, 3.17)	0.120
Potassium, mmol/L	4.37 ± 0.70	4.32 ± 0.61	0.138
Sodium, mmol/L	140.00 ± 6.68	139.78 ± 5.57	0.386
BUN, mmol/L	25.00 (17.00, 36.00)	21.00 (14.00, 31.00)	<0.001
Creatinine, mg/dL	1.18 (0.81, 1.57)	1.00 (0.80, 1.33)	<0.001
WBC, 10^9/L	15.80 (11.10, 22.00)	15.90 (11.00, 21.40)	0.669
Platelet, 10^9/L	178.00 (131.00, 250.00)	194.50 (139.00, 260.00)	0.005
INR	1.27 (1.13, 1.47)	1.20 (1.11, 1.40)	0.001
PT, s	15.06 (13.70, 17.00)	14.85 (13.73, 16.64)	0.242
Heart rate(beats per min)	93.35 (84.51, 104.00)	93.58 (83.02, 105.44)	0.790
Respiratory rate(breaths per min)	21.01 (18.67, 23.61)	21.07 (18.76, 23.89)	0.418
SpO_2,_ %	96.67 (95.68, 98.00)	96.49 (95.16, 97.75)	<0.001
SBP, mmHg	113.80 ± 16.09	113.80 ± 15.69	0.781
DBP, mmHg	62.51 ± 9.93	63.26 ± 9.92	0.068
Temperature, ℃	37.03 ± 0.65	37.09 ± 0.66	0.048

Data on sex were missing for one patient in the UFH group.

UFH, Unfractionated heparin; BMI, Body Mass Index; Hb, hemoglobin; BUN, blood urea nitrogen; WBC, White Blood Cell; INR, international normalized ratio; PT, Prothrombin time; SpO_2_, percutaneous arterial oxygen saturation; SBP, Systolic blood pressure; DBP, Diastolic blood pressure.

Albumin, Hb, Potassium, Sodium, SBP, DBP, Temperature:Mean ± SD.

Age, BMI, APACHE IV, Bilirubin, Lactate, BUN, Creatinine, WBC, Platelet, INR, PT, Heart rate, Respiratory rate, SpO_2_:median (Q1, Q3).

### Survival analysis

Kaplan–Meier curves (Figure 2) showed that the enoxaparin group had numerically lower mortality rates than the UFH group at both 30 days in the ICU and 90 days in the hospital. However, the differences were not statistically significant (log-rank *P* = 0.337 and 0.119, respectively).

**Figure 2:**
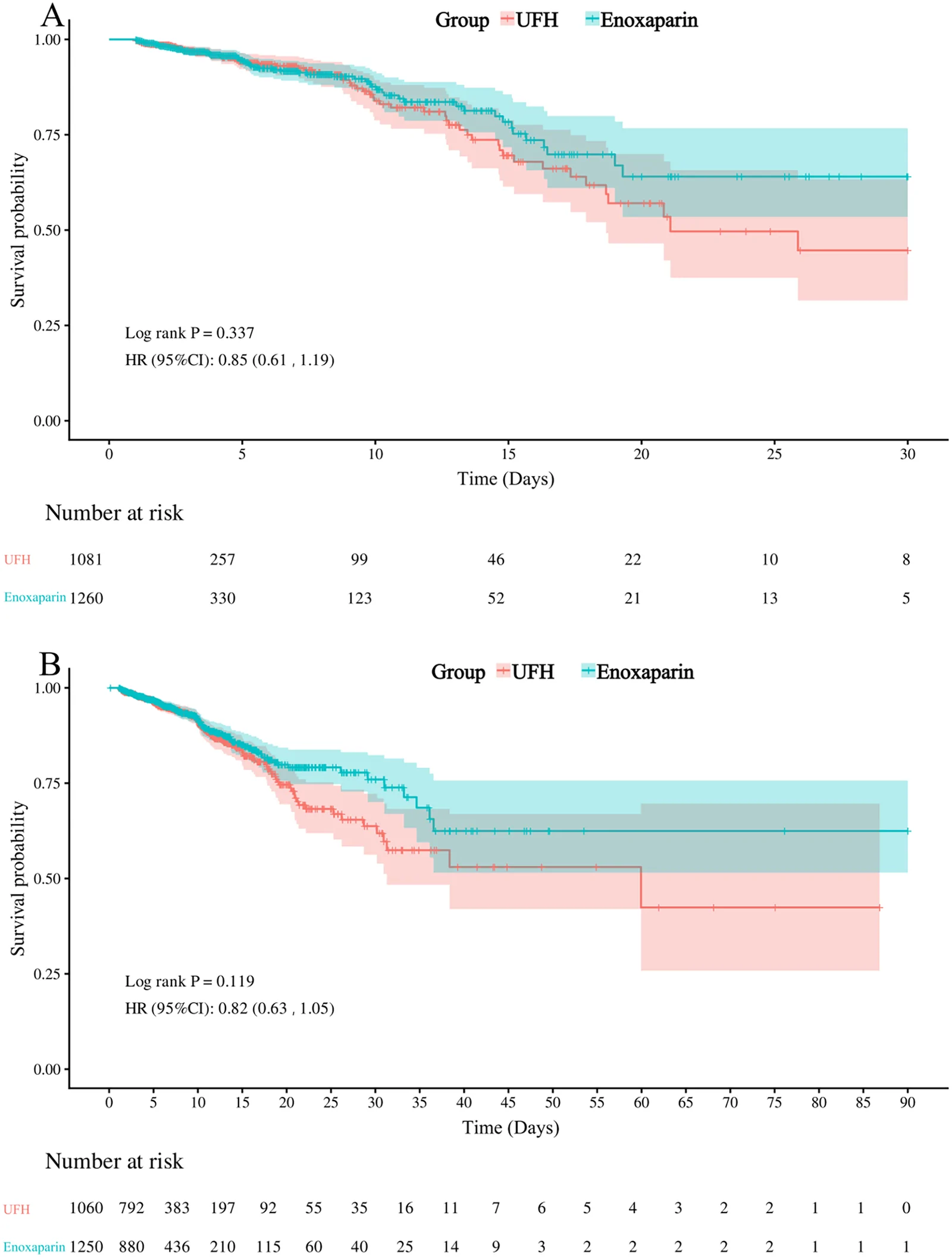
**(A)** Kaplan–Meier survival curves for sepsis patients with enoxaparin or UFH at 30 days in ICU. **(B)** Kaplan–Meier survival curves for sepsis patients with enoxaparin or UFH at 90 days in hospital. The total number of at risk patients are shown below for each time point.

### Adjusted analysis of mortality risk

The association between anticoagulant type and mortality was further assessed using multivariable Cox proportional-hazards regression (Table 2). In the unadjusted analysis (Model 1), the HR for enoxaparin versus UFH was 0.85 (95% CI: 0.61–1.19; *P* = 0.337) for 30-day ICU mortality and 0.82 (95% CI: 0.63–1.05; *P* = 0.119) for 90-day in-hospital mortality. After adjustment for all covariates (Model 3), the HR for 30-day ICU mortality was 0.94 (95% CI: 0.67–1.32; *P* = 0.726) and for 90-day in-hospital mortality was 0.86 (95% CI: 0.66–1.12; *P* = 0.271).

**Table 2:** Multivariate Cox regression analysis.

Outcome	Group	Model 1		Model 2		Model 3	
		HR (95%CI)	*P*-value	HR (95%CI)	*P*-value	HR (95%CI)	*P*-value
30 days in-ICU mortality	UFH	1.00 (Reference)		1.00 (Reference)		1.00 (Reference)	
	Enoxaparin	0.85 (0.61, 1.19)	0.337	0.90 (0.64, 1.25)	0.519	0.94 (0.67, 1.32)	0.726
90 days in-hospital mortality	UFH	1.00 (Reference)		1.00 (Reference)		1.00 (Reference)	
	Enoxaparin	0.82 (0.63, 1.05)	0.119	0.87 (0.67, 1.12)	0.278	0.86 (0.66, 1.12)	0.271

HR, Hazard Ratio; CI, Confidence Interval.

Model1: Crude.

Model2: Adjust: Sex, Age, BMI.

Model3: Adjust: Sex, Age, BMI, APACHE IV, Albumin, Bilirubin, Creatinine, Lactate, BUN, SpO_2_, Heart rate.

### Subgroup analysis

Forest plots of subgroup analyses (Figure 3) evaluated the effect of enoxaparin versus UFH on mortality stratified by sex, age, and BMI. For both 30-day ICU mortality and 90-day in-hospital mortality, no statistically significant interactions were observed across any of the examined subgroups (all P for interaction >0.05), suggesting that the association between anticoagulant type and mortality was not modified by sex, age, or BMI within this cohort.

**Figure 3:**
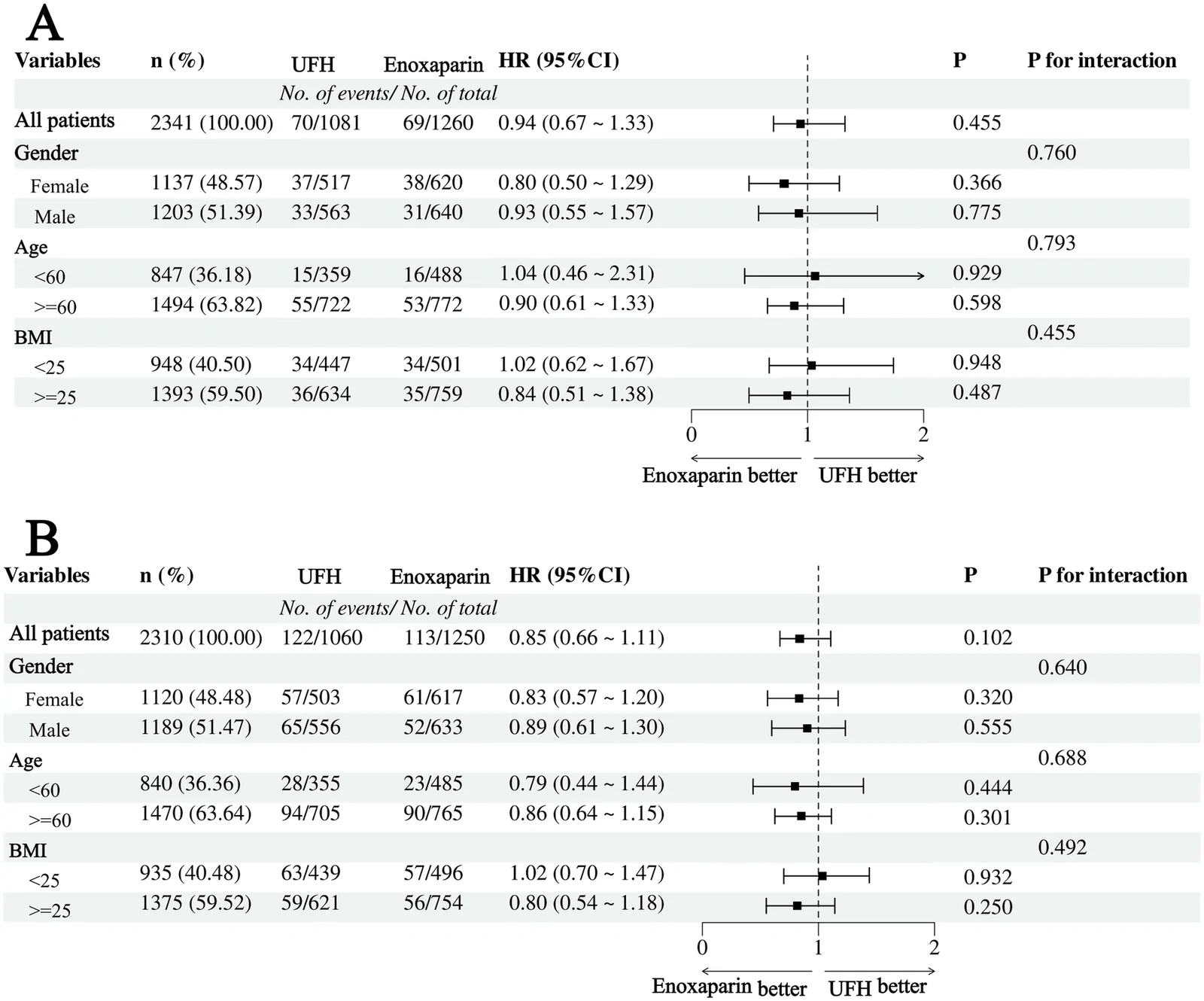
**(A)** Forest plot analysis of mortality risk patients receiving enoxaparin compared with UFH at 30 days in ICU. **(B)** Forest Plot Analysis of Mortality Risk Patients Receiving Enoxaparin Compared with UFH at 90 days in hospital. Data on sex were missing for one patient in the UFH group.

### Sensitivity analysis

To validate the robustness of the primary findings, a sensitivity analysis using IPTW was performed on all 2,341 patients (Table 3). After weighting, all SMDs were below 0.1 (Supplementary Tables S1, S2; Supplementary Figures S1, S2), indicating good balance of baseline covariates between the two groups. The weighted HR for enoxaparin versus UFH was 0.91 (95% CI: 0.64-1.27; *P*=0.578) for 30-day ICU mortality and 0.88 (95% CI: 0.68–1.14; *P* = 0.331) for 90-day in-hospital mortality. Weighted Kaplan–Meier curves (Supplementary Figure S3) showed no significant difference between groups, consistent with the primary unadjusted analysis.

**Table 3:** Multivariate Cox regression analysis after inverse probability of treatment weighting.

Outcome	Group	HR (95%CI)	*P*-value
30 days in-ICU mortality	UFH	1.0	
	Enoxaparin	0.91 (0.65, 1.27)	0.578
90 days in-hospital mortality	UFH	1.0	
	Enoxaparin	0.88 (0.68, 1.14)	0.331

Adjusted for: Sex, Age, BMI, APACHE IV, Albumin, Bilirubin, Creatinine, Lactate, BUN, SpO_2_, Heart rate.

## Discussion

This analysis of the eICU Collaborative Research Database found that, among 2,341 sepsis patients without severe renal impairment, the point estimates for enoxaparin versus UFH were consistently below 1.0 across all models for both 30-day ICU mortality (adjusted HR = 0.94, 95% CI: 0.67–1.32; *P* = 0.726) and 90-day in-hospital mortality (adjusted HR = 0.86, 95% CI: 0.66–1.12; *P* = 0.271). The corresponding IPTW analyses yielded similar estimates (30-day: HR = 0.91, 95% CI: 0.64–1.27; 90-day: HR = 0.88, 95% CI: 0.68–1.14). Although these differences did not reach statistical significance—and the wide confidence intervals indicate that both clinically meaningful benefit and negligible effect remain compatible with the data—the direction and consistency of the point estimates across analytical approaches raise the possibility that enoxaparin may confer a modest survival advantage in this population. Subgroup analyses showed no significant heterogeneity across sex, age, and BMI strata (all P for interaction >0.05).

Several studies have reported mortality benefits of heparin therapy in sepsis. UFH was associated with reduced 28-day mortality in adult sepsis patients (HR 0.70, 95% CI 0.56–0.87) (8–10), and LMWH was identified as an independent protective factor against 28-day mortality in a sepsis-3 cohort (HR 0.48, 95% CI 0.29–0.81) (11). However, these findings derive from comparisons against placebo or no heparin, rather than direct head-to-head comparisons between UFH and LMWH. Moreover, many of these studies included patients with varying degrees of renal function, introducing potential confounding due to the differential renal clearance profiles of the two heparins (17–19).. Consequently, whether enoxaparin confers a mortality advantage over UFH in sepsis patients with preserved renal function has remained unknown.

In contrast to previous studies, the present study specifically focused on sepsis patients without severe renal impairment (eGFR ≥ 30 mL/min/1.73 m^2^)—a population in which the comparative efficacy of enoxaparin versus UFH has not been previously defined. By excluding patients with significant renal dysfunction, we minimized the confounding effect of differential drug accumulation, wherein enoxaparin—being renally cleared—would be expected to accumulate and increase bleeding risk in patients with impaired renal function (15, 20, 21). This design allowed a more direct comparison of the two agents’ intrinsic effects on mortality, independent of the major pharmacokinetic confounder that has complicated prior observational comparisons.

The observed HRs—0.94 for 30-day ICU mortality and 0.86 for 90-day in-hospital mortality—demonstrate a consistent trend toward lower mortality with enoxaparin compared with UFH. While the confidence intervals cross 1.0 and the *P* values do not reach conventional statistical significance, the magnitude of these estimates warrants clinical attention. A 15% relative risk reduction at 90 days, if confirmed in future studies, would represent a clinically meaningful benefit in the sepsis population, given the persistently high baseline mortality rates (exceeding 25% globally) (22, 23), and the widespread routine use of prophylactic anticoagulation in ICUs. Moreover, the consistent direction of effect across both time points (30 and 90 days) and across all subgroups examined—with no meaningful heterogeneity by age, sex, or BMI—strengthens the plausibility that this trend reflects a genuine biological signal rather than chance variation. The fact that the IPTW-adjusted estimates (HR = 0.91 and 0.88) closely align with the primary adjusted analyses further supports this interpretation.

The exclusion of patients with severe renal impairment is particularly informative in this context. In patients with significant renal dysfunction, enoxaparin clearance is impaired, leading to drug accumulation, supratherapeutic anti-Xa levels, and an increased risk of major bleeding (15, 21)—a complication that could offset any mortality benefit the drug might otherwise provide. By restricting our analysis to patients with preserved renal function, we removed this major safety concern and created the pharmacokinetic condition most favorable for detecting a potential advantage of enoxaparin over UFH. The finding of a consistent mortality trend favoring enoxaparin in this carefully selected population suggests that the drug's potential benefit is not obscured by bleeding complications related to accumulation, but rather reflects a genuine pharmacologic effect. Furthermore, although patients in the enoxaparin group had more favorable baseline characteristics (younger age, lower APACHE IV scores, and better renal function), multivariable adjustment attenuated—but did not reverse—the direction of effect, with the HR moving from 0.85 (unadjusted) to 0.94 (adjusted) for 30-day mortality and remaining below 1.0 for both outcomes. This further supports the robustness of the observed trend against confounding. Several factors may explain why this trend did not reach statistical significance despite its consistency. First, the magnitude of the true difference between the two active anticoagulants is likely modest—detecting a 10%–15% relative risk reduction in a head-to-head comparison of two active agents requires substantially larger sample sizes than studies comparing heparin against placebo. Second, by excluding patients with severe renal impairment, our cohort had a relatively lower event rate than studies that included higher-risk subgroups such as those with AKI or coagulopathy (17–19), further limiting statistical power. Third, the non-anticoagulant properties of UFH—including anti-inflammatory effects and inhibition of Drp1-mediated mitochondrial dysfunction (24)—may provide ancillary benefits in sepsis that partially offset the pharmacokinetic advantages of enoxaparin, particularly in patients without renal clearance impairment, thereby narrowing the expected mortality difference between the two agents. Taken together, these factors suggest that the lack of statistical significance does not negate the potential clinical relevance of the observed trend; rather, it reflects the inherent challenge of detecting a modest effect in a comparative-effectiveness study of two active therapies.

The present study offers several methodological strengths. First, by specifically focusing on sepsis patients without severe renal impairment, we controlled for a key pharmacokinetic confounder—a major barrier to valid head-to-head comparisons in prior observational studies (25–27). This design enhances the internal validity of the comparison between enoxaparin and UFH. Second, the use of a large-scale, real-world retrospective cohort enhances the clinical representativeness and generalizability of the findings, reflecting routine clinical practice across diverse U.S. hospitals. Third, the robustness of the primary results was rigorously evaluated through comprehensive sensitivity analyses, including subgroup and IPTW analyses, which consistently supported the main findings and reduced concerns about residual confounding from measured covariates.

However, this study has several limitations. First, owing to the limited number of outcome events, the adjustment models could not incorporate all potentially relevant confounders. Variables such as the SOFA score, DIC/SIC severity scores, vasopressor use, and mechanical ventilation were not included in the adjusted models. Additionally, data on pre-existing comorbidities (e.g., diabetes, hypertension), bleeding risk, infection source, and corticosteroid therapy were not adjusted for. The omission of these factors may have introduced residual confounding and could bias the estimated treatment effects. Notably, the original database precluded adjustment for anticoagulation duration—a known prognostic factor (28, 29)—which may have biased the effect size in either direction. Second, our analysis focused exclusively on mortality and did not evaluate other clinically relevant endpoints, including major bleeding, transfusion requirements, DVT, PE, HIT, and length of stay. Data on HIT were not systematically recorded in the database and could not be retrieved. For the remaining endpoints, they were not captured in the present analysis. Consequently, a formal assessment of net clinical benefit could not be performed. Third, sepsis cases were identified using ICD-10 discharge diagnosis codes, which reflect clinical diagnoses made prior to the adoption of the Sepsis-3 definitions; consequently, the study population may not fully overlap with a cohort defined by prospective application of Sepsis-3 criteria, potentially resulting in some degree of misclassification. Fourth, the database does not record the clinical rationale for selecting UFH versus LMWH. In exploratory analyses, greater disease severity and lower albumin were associated with a higher likelihood of receiving enoxaparin (Supplementary Table S3). Although we restricted the cohort to patients with eGFR ≥ 30 mL/min/1.73m^2^ and adjusted for sex, age, BMI, APACHE IV, albumin, bilirubin, creatinine, lactate, BUN, heart rate, and SpO_2_ in both Cox and IPTW models, which partially reduced baseline imbalances (Supplementary Table S4), residual confounding by indication may remain. In addition, this study compared only enoxaparin with UFH, as no other LMWHs were recorded in the eICU-CRD. Because different LMWHs differ in molecular weight, anti-Xa/anti-IIa activity ratio, half-life, and renal clearance, the present findings should not be extrapolated to other LMWHs. Whether other LMWHs differ from UFH or from each other in sepsis remains unknown and warrants future investigation. Finally, the sample was predominantly from U.S. White patients, which may limit generalizability to other racial/ethnic groups and healthcare settings outside the United States.

## Conclusions

In summary, in sepsis patients without severe renal impairment, enoxaparin showed a consistent but not statistically significant trend toward lower mortality compared with UFH (HR = 0.85–0.94). The effect size is clinically meaningful, and the consistency across multiple analytical approaches supports a genuine though modest benefit. Future adequately powered randomized trials incorporating both thrombotic and bleeding outcomes are warranted to confirm whether enoxaparin confers a net advantage over UFH in this population.

## Data Availability

Publicly available datasets were analyzed in this study. This data can be found here: https://eicu-crd.mit.edu.

## Supplementary material

Supplementary Figure S1 Love plot displaying SMDs before and after IPTW weighting.

Supplementary Figure S2 Propensity score overlap.

Supplementary Figure S3 IPTW-adjusted Kaplan–Meier survival curves for septic patients receiving enoxaparin versus UFH. **(A)** 30-day ICU mortality. **(B)** 90-day in-hospital mortality.

Supplementary Table S1 SMDs before and after weighting.

Supplementary Table S2 Weighted baseline balance.

Supplementary Table S3 Multivariable logistic regression analysis of factors associated with the choice of enoxaparin versus UFH in the full cohort.

Supplementary Table S4 Baseline characteristics before and after restricting the cohort to patients with eGFR ≥30 mL/min/1.73 m².
