# Supplementary material for: Comparative effectiveness of enoxaparin versus unfractionated heparin for 30- and 90-day mortality in sepsis patients without severe renal dysfunction: a retrospective cohort study
Source: Front Med (Lausanne). 2026 Oct 26;13:1886512. doi: 10.3389/fmed.2026.1886512 (PMC13745486; doi:10.3389/fmed.2026.1886512)
Supplement: Supplementary file 1 [file Table1.docx]

**Table S 1：**SMDs before and after weighting

The dashed vertical line indicates the threshold of SMD = 0.1. Squares represent SMDs before weighting, and circles represent SMDs after weighting. All covariates achieved SMD < 0.1 after IPTW, indicating successful balance.

| Label | SMD Before | SMD After |
| --- | --- | --- |
| Age | -0.2215 | -0.0048 |
| Gender | -0.0244 | 0.0039 |
| BMI | 0.0301 | 0.0039 |
| APACHE IV | -0.1119 | -0.0021 |
| Albumin | -0.0501 | 0.0006 |
| Bilirubin | -0.0913 | -0.0133 |
| Creatinine | -0.28 | -0.0023 |
| SpO2 | -0.1427 | -0.0022 |
| INR | 0.0075 | -0.0019 |
| Lactate | -0.0301 | -0.0006 |
| BUN | -0.2933 | -0.0143 |
| Heart Rate | 0.0063 | -0.0006 |
