# Supplementary material for: Comparative effectiveness of enoxaparin versus unfractionated heparin for 30- and 90-day mortality in sepsis patients without severe renal dysfunction: a retrospective cohort study
Source: Front Med (Lausanne). 2026 Oct 26;13:1886512. doi: 10.3389/fmed.2026.1886512 (PMC13745486; doi:10.3389/fmed.2026.1886512)
Supplement: Supplementary file 2 [file Table2.docx]

**Table S2:** Weighted baseline balance.

| Variable | UFH | LWMH | p | SMD |
| --- | --- | --- | --- | --- |
| Age mean (SD) | 63.58 (17.52) | 63.49 (16.98) | 0.912 | -0.0048 |
| Gender (%) |  |  | 0.928 | 0.0039 |
| Female | 48.84% | 48.64% |  |  |
| Male | 51.16% | 51.36% |  |  |
| BMI mean (SD) | 28.41 (9.36) | 28.44 (8.96) | 0.928 | 0.0039 |
| APACHE IV mean (SD) | 63.68 (22.93) | 63.63 (22.53) | 0.961 | -0.0021 |
| Albumin mean (SD) | 2.7 (0.66) | 2.7 (0.66) | 0.989 | 0.0006 |
| Bilirubin mean (SD) | 1.08 (1.53) | 1.06 (1.42) | 0.757 | -0.0133 |
| Creatinine mean (SD) | 1.14 (0.48) | 1.14 (0.44) | 0.957 | -0.0023 |
| SpO_2_ mean (SD) | 96.52 (2.08) | 96.51 (1.89) | 0.961 | -0.0022 |
| INR mean (SD) | 1.43 (0.96) | 1.43 (0.92) | 0.966 | -0.0019 |
| Lactate mean (SD) | 2.7 (1.93) | 2.7 (1.88) | 0.990 | -0.0006 |
| BUN mean (SD) | 26.49 (15.95) | 26.27 (15.35) | 0.740 | -0.0143 |
| Heart rate mean (SD) | 94.25 (15.81) | 94.24 (16.36) | 0.988 | -0.0006 |
