# Supplementary material for: Comparative effectiveness of enoxaparin versus unfractionated heparin for 30- and 90-day mortality in sepsis patients without severe renal dysfunction: a retrospective cohort study
Source: Front Med (Lausanne). 2026 Oct 26;13:1886512. doi: 10.3389/fmed.2026.1886512 (PMC13745486; doi:10.3389/fmed.2026.1886512)
Supplement: Supplementary file 3 [file Table3.docx]

**Table S3:** Multivariable logistic regression analysis of factors associated with the choice of enoxaparin versus UFH in the full cohort.

| N=3350 | OR | coefficient | 95%CI.low | 95%CI.upp | P-value |
| --- | --- | --- | --- | --- | --- |
| Age | 1.03 | 0.03 | 1.02 | 1.04 | < 0.001 |
| Sex (Male vs Female) | 1.00 | 0.00 | 0.79 | 1.25 | 0.971 |
| BMI, kg/m² | 1.00 | 0.00 | 1.00 | 1.00 | 0.645 |
| APACHE IV | 1.01 | 0.01 | 1.01 | 1.02 | < 0.001 |
| Albumin, g/dL | 0.53 | -0.63 | 0.44 | 0.65 | < 0.001 |
| Bilirubin, mg/dL | 1.07 | 0.07 | 1.02 | 1.13 | 0.004 |
| Creatinine, mg/dL | 0.98 | -0.02 | 0.90 | 1.07 | 0.669 |
| Lactate, mmol/L | 1.11 | 0.10 | 1.06 | 1.16 | < 0.001 |
| BUN, mmol/L | 1.00 | 0.00 | 0.99 | 1.00 | 0.656 |
| Heart rate | 1.02 | 0.02 | 1.01 | 1.03 | < 0.001 |
| SpO₂, mmHg | 0.88 | -0.13 | 0.84 | 0.93 | < 0.001 |
| PT, s | 1.02 | 0.02 | 1.01 | 1.03 | < 0.001 |
