# Supplementary material for: Comparative effectiveness of enoxaparin versus unfractionated heparin for 30- and 90-day mortality in sepsis patients without severe renal dysfunction: a retrospective cohort study
Source: Front Med (Lausanne). 2026 Oct 26;13:1886512. doi: 10.3389/fmed.2026.1886512 (PMC13745486; doi:10.3389/fmed.2026.1886512)
Supplement: Supplementary file 4 [file Table4.docx]

|  | Total( n = 3350) | | | eGFR ≥ 30 ( n = 2341) | | |
| --- | --- | --- | --- | --- | --- | --- |
| Variable | UFH(n = 1886) | Enoxaparin(n = 1464) | P-value | UFH(n = 1081) | Enoxaparin(n = 1260) | P-value |
| Age | 68.00 (56.00, 80.00) | 65.00 (53.00, 75.00) | <0.001 | 67.00 (55.00, 79.00) | 64.00 (52.00, 74.00) | <0.001 |
| Sex, n (%) |  |  | 0.799 |  |  | 0.465 |
| Male | 956 (50.72) | 736 (50.27) |  | 517 (47.83) | 620 (49.21) |  |
| Female | 929 (49.28) | 728 (49.73) |  | 563 (52.08) | 640 (50.79) |  |
| BMI, kg/m² | 27.06 (22.71, 32.82) | 27.12 (22.70, 33.07) | 0.964 | 26.41 (22.35, 31.77) | 26.85 (22.37, 32.67) | 0.294 |
| APACHE IV | 70.00 (55.00, 85.00) | 63.00 (49.00, 78.00) | <0.001 | 63.49 (49.55, 78.00) | 61.00 (47.00, 75.66) | 0.004 |
| Albumin, g/dL | 2.63 ± 0.65 | 2.65 ± 0.66 | 0.473 | 2.71 ± 0.66 | 2.68 ± 0.67 | 0.223 |
| Bilirubin, mg/dL | 0.74 (0.50, 1.20) | 0.70 (0.50, 1.10) | 0.005 | 0.73 (0.50, 1.12) | 0.70 (0.50, 1.10) | 0.113 |
| Hb, g/dL | 9.96 ± 2.09 | 10.54 ± 2.18 | <0.001 | 10.26 ± 2.10 | 10.62 ± 2.19 | <0.001 |
| Lactate, mmol/L | 2.40 (1.60, 3.50) | 2.30 (1.50, 3.30) | 0.048 | 2.31 (1.60, 3.30) | 2.21 (1.50, 3.17) | 0.120 |
| Potassium, mmol/L | 4.59 ± 0.90 | 4.39 ± 0.67 | <0.001 | 4.37 ± 0.70 | 4.32 ± 0.61 | 0.138 |
| Sodium, mmol/L | 140.06 ± 6.87 | 139.84 ± 5.82 | 0.316 | 140.00 ± 6.68 | 139.78 ± 5.57 | 0.386 |
| BUN, mmol/L | 36.00 (22.00, 56.00) | 23.00 (16.00, 35.00) | <0.001 | 25.00 (17.00, 36.00) | 21.00 (14.00, 31.00) | <0.001 |
| Creatinine, mg/dL | 1.80 (1.09, 3.13) | 1.10 (0.82, 1.60) | <0.001 | 1.18 (0.81, 1.57) | 1.00 (0.80, 1.33) | <0.001 |
| WBC, 10^9/L | 16.20 (11.50, 22.54) | 16.20 (11.30, 21.90) | 0.498 | 15.80 (11.10, 22.00) | 15.90 (11.00, 21.40) | 0.669 |
| Platelet, 10^9/L | 176.00 (127.00, 247.00) | 193.00 (136.00, 259.00) | 0.001 | 178.00 (131.00, 250.00) | 194.50 (139.00, 260.00) | 0.005 |
| INR | 1.30 (1.15, 1.50) | 1.22 (1.12, 1.42) | <0.001 | 1.27 (1.13, 1.47) | 1.20 (1.11, 1.40) | 0.001 |
| PT, s | 15.43 (13.81, 17.71) | 14.99 (13.80, 17.00) | <0.001 | 15.06 (13.70, 17.00) | 14.85 (13.73, 16.64) | 0.242 |
| Heart rate | 92.09 (82.00, 103.02) | 93.53 (83.00, 105.40) | 0.021 | 93.35 (84.51, 104.00) | 93.58 (83.02, 105.44) | 0.790 |
| Respiratory rate | 20.66 (18.32, 23.33) | 21.08 (18.76, 23.90) | 0.004 | 21.01 (18.67, 23.61) | 21.07 (18.76, 23.89) | 0.418 |
| SpO_2_, mmHg | 96.91 (95.80, 98.22) | 96.50 (95.14, 97.76) | <0.001 | 96.67 (95.68, 98.00) | 96.49 (95.16, 97.75) | <0.001 |
| SBP, mmHg | 112.70 ± 16.34 | 113.40 ± 15.95 | 0.213 | 113.80 ± 16.09 | 113.80 ± 15.69 | 0.781 |
| DBP, mmHg | 60.85 ± 10.26 | 62.78 ± 10.05 | <0.001 | 62.51 ± 9.93 | 63.26 ± 9.92 | 0.068 |
| Temperature, ℃ | 36.95 ± 0.65 | 37.07 ± 0.67 | <0.001 | 37.03 ± 0.65 | 37.09 ± 0.66 | 0.048 |

**Table S4:** Baseline characteristics before and after restricting the cohort to patients with eGFR ≥30 mL/min/1.73 m².

*UFH=Unfractionated heparin; BMI=Body Mass Index; Hb=hemoglobin; BUN=blood urea nitrogen; WBC=White Blood Cell; INR=international normalized ratio; PT=Prothrombin time; SpO_2_=percutaneous arterial oxygen saturation; SBP=Systolic blood pressure; DBP=Diastolic blood pressure*

*Albumin, Hb, Potassium, Sodium, SBP, DBP, Temperature:Mean±SD*

*Age, BMI, APACHE IV, Bilirubin, Lactate, BUN, Creatinine, WBC, Platelet, INR, PT, Heart rate, Respiratory rate, SpO_2_:median (Q1, Q3)*
